# A ketogenic diet enhances aerobic exercise adaptation and promotes muscle mitochondrial remodeling in hyperglycemic mice

**DOI:** 10.21203/rs.3.rs-5814971/v1

**Published:** 2025-01-29

**Authors:** Pattarawan Pattamaprapanont, Roberto C. Nava, Mia Formato, Eileen M. Cooney, Ana Paula Pinto, Ana B. Alves-Wagner, Anamica Das, Yuntian Guan, Sarah J. Lessard

**Affiliations:** 1 Research Division; Joslin Diabetes Center, Boston, MA, USA.; 2 Harvard Medical School, Boston, MA, USA.; 3 Center for Exercise Medicine Research; Fralin Biomedical Research Institute at Virginia Tech Carilion, Roanoke, VA, USA.; 4 Department of Human Nutrition, Foods, and Exercise; Virginia Polytechnic Institute and State University, Blacksburg, VA, USA.

## Abstract

VO_2_peak is a key health benefit of aerobic exercise; however, chronic hyperglycemia is associated with persistently low VO_2_peak due to an impaired adaptive response to training. Here, we tested whether reducing blood glucose with a low-carbohydrate/high-fat “ketogenic” diet could restore aerobic exercise adaptation in a mouse model of hyperglycemia. Hyperglycemia was induced by streptozotocin (STZ) and mice were stratified to standard chow (STZ-CHOW), or a ketogenic diet (STZ-KETO), which rapidly normalized blood glucose. After aerobic exercise training, improvements in VO_2_peak were blunted in STZ-CHOW, but exercise response was restored in STZ-KETO. Improved VO_2_peak in STZ-KETO was associated with enhanced aerobic remodeling of skeletal muscle, including a more oxidative fiber-type and increased capillary density, along with restoration of circulating angiogenic markers. Moreover, KETO induced exercise-independent effects on muscle mitochondrial remodeling and mitochondrial dynamics, significantly increasing fatty acid oxidation. Our results identify a ketogenic diet as a potential therapy to improve aerobic exercise response in the growing population with hyperglycemia due to diabetes and other metabolic conditions.

Data from humans and animal models demonstrate that chronic hyperglycemia is associated with a blunted adaptive response to aerobic exercise training^1–3^. A key exercise adaptation that is negatively impacted in those with hyperglycemia is improved aerobic exercise capacity, measured as maximal or peak oxygen consumption rate (i.e. VO_2_max or VO_2_peak)^4–7^. High aerobic exercise capacity is one of the strongest predictors of health and longevity in preclinical and clinical studies^8–10^. Moreover, in people with diabetes, maintaining a high exercise capacity is associated with lower risk of complications such as cardiovascular and kidney disease^11–15^. Given the strong association between hyperglycemia and impaired aerobic adaptation, strategies to reduce blood glucose, when combined with aerobic training, may enhance the adaptive response to exercise.

Ketogenic diets are characterized by high fat (≥60% [kcal]) and very low carbohydrate (<10% [kcal], or <50 g/day) content, which reduces glucose availability and induces production of ketone bodies by the liver^16^. Prior to the discovery and availability of insulin in the 1920s, ketogenic diets were a foundation for the treatment of type 1 diabetes due to their efficacy in lowering blood glucose concentrations, thereby promoting longer survival in the face of insulin deficiency^17^. Ketogenic diets have also proven to be therapeutically beneficial for the treatment of several neurological conditions, including epilepsy^18^. However, whether consumption of high-fat, low-carbohydrate diets can benefit the general population is controversial due to evolving views regarding the contribution of dietary fat to obesity and metabolic disease^19^.

Recently, interest has reemerged for the use of ketogenic diets to promote weight loss and enhance athletic performance^19–22^. Mounting evidence demonstrates potential for ketogenic diets to improve glucose homeostasis and reduce body weight in those with type 2 diabetes^22–24^. In people with type 1 diabetes, ketogenic diets may reduce the required daily insulin dose and allow for maintenance of HbA1c at near normal levels (<6%)^25, 26^. With respect to improving exercise performance, meta-analysis of published data demonstrates that ketogenic diets do not appear to have beneficial or harmful effects in young, healthy individuals without metabolic disease^21, 27^. However, whether ketogenic diets can improve exercise adaptation and aerobic capacity in the growing population of individuals with hyperglycemia^28, 29^ due to type 1 diabetes, type 2 diabetes, or other metabolic disorders is a key unanswered question.

Our previous work demonstrates that mice with hyperglycemia due to low-dose streptozotocin (STZ) treatment have blunted improvements in VO_2_peak with training and impaired skeletal muscle remodeling with exercise^1, 2^. Here, we tested the hypothesis that feeding STZ-treated mice a ketogenic diet would reduce blood glucose, leading to an improved adaptive response to voluntary wheel running. Indeed, we demonstrate that a diet consisting of 90% fat and 10% protein (KETO) can normalize blood glucose in STZ-treated mice. Moreover, KETO restored improvements in VO_2_peak and enhanced muscle remodeling in response to aerobic training in STZ. KETO also induced exercise-independent changes in skeletal muscle fiber type and mitochondrial remodeling. Our data identify a low-carbohydrate/high fat diet as a potential therapy to improve exercise response in diabetes.

## RESULTS

### Metabolic effects of a ketogenic diet.

**Figure 1.**
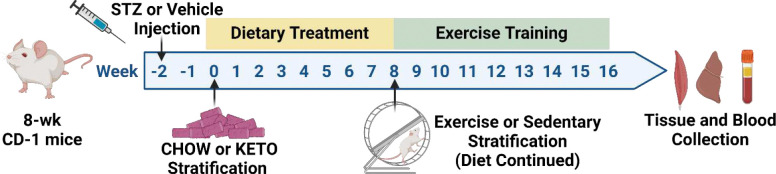
Experimental design and timeline. 8-wk old male CD-1 mice were injected on two consecutive days with 40 mg/kg streptozotocin to induce hyperglycemia. Two weeks later, hyperglycemic mice were stratified to receive a typical high carbohydrate diet (CHOW), or a high-fat ketogenic diet (KETO). 8-wks after diet induction, mice were stratified into static cages (Sedentary), or voluntary wheel running cages (Exercise Training). Original diets were maintained during the exercise-training period. Following 8-wks of exercise, blood and tissues were collected. Figure was created using Biorender.com.

**Figure 2.**
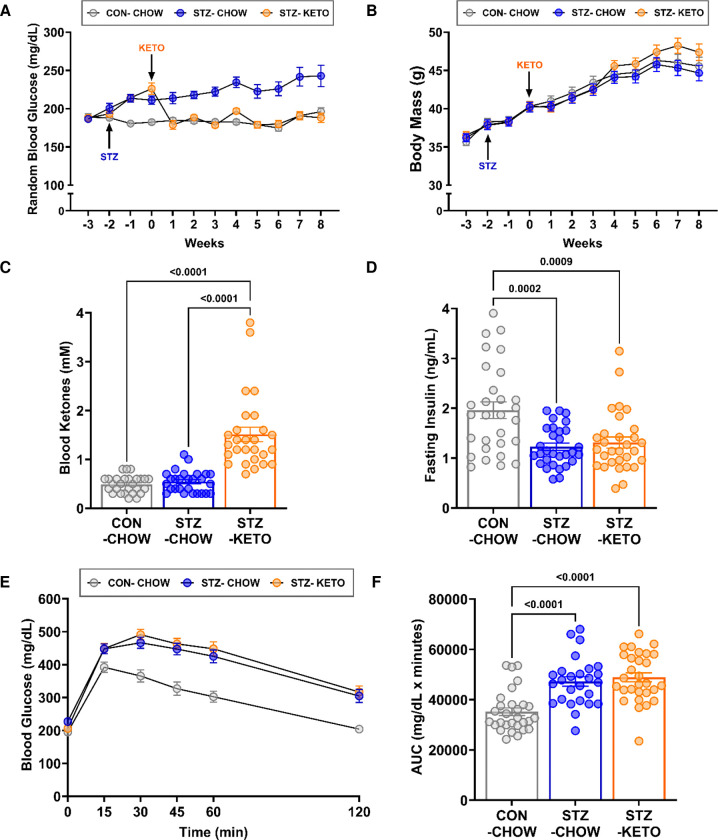
A ketogenic diet reverses STZ-induced hyperglycemia. **[A]** Male CD-1 mice were injected with 2 doses of 40mg/kg streptozotocin (STZ), or vehicle (CON) at week 0. After development of hyperglycemia, mice were stratified to receive a control high-carbohydrate diet (CHOW) or a ketogenic diet (KETO). Blood glucose was measured weekly. **[B]** Body mass was similar among groups for the initial 8-wk feeding period. **[C]** Blood ketones were significantly elevated in KETO compared to CHOW-fed groups. **[D]** Fasting insulin was ~50% lower in both STZ-treated groups, independent of dietary treatment. **[E]** Glucose tolerance was impaired in both STZ-treated groups, independent of dietary treatment, **[F]** as demonstrated by higher glucose area under the curve. Results are displayed as mean ± SEM. One-way ANOVA with Tukey post-hoc testing was used to determine differences among groups.

This study aimed to test the hypothesis that glucose reduction via consumption of a ketogenic diet in hyperglycemic mice can restore aerobic adaptation to exercise training. Figure 1 outlines our experimental design and timeline. We first tested the metabolic effects of a ketogenic diet on mice with hyperglycemia (Figure 2). Hyperglycemia was induced using low dose (40 mg/kg) intraperitoneal injections of streptozotocin (STZ) for two consecutive days, and was defined as blood glucose of more than 200 mg/dL. Two-weeks following STZ injection, hyperglycemic mice were stratified to receive regular chow (STZ-CHOW), or a ketogenic diet (STZ-KETO). Normoglycemic mice (vehicle-injected) consuming regular chow (CON-CHOW) served as controls.

The ketogenic diet normalized blood glucose in STZ-treated mice to the level of CON-CHOW within 1-week of feeding, while STZ-CHOW continued to exhibit hyperglycemia (Figure 2A). Throughout the initial 8-week dietary treatment period, blood glucose in STZ-KETO was sustained within the normoglycemic range without developing hypoglycemia. Neither STZ injection or KETO altered body mass during the first 8-weeks of treatment (Figure 2B). After 8 weeks of diet, fasting blood ketones were measured, and ketonemia was observed in STZ-KETO (Figure 2C), demonstrating the efficacy of our dietary paradigm. However, KETO did not alter STZ-induced hypoinsulinemia (Figure 2D) or glucose intolerance (Figure 2E and 2F). These data demonstrate that a ketogenic diet can effectively maintain blood glucose levels of STZ-treated mice in the normoglycemic range, despite low circulating insulin and glucose intolerance.

Prior to beginning the exercise-training intervention, we determined if diet alone can impact aerobic capacity in hyperglycemic mice by performing maximal exercise testing after 8-weeks of dietary treatment. In sedentary mice, there were no significant effects of hyperglycemia or diet on VO_2_peak (Supplementary Figure 1A) or time to exhaustion (Supplementary Figure 1B). The observation that hyperglycemia does not impact aerobic capacity in the absence of training is consistent with our previous studies^1, 2^. These data also demonstrate similar starting aerobic capacity among groups, which is important for interpretation of exercise-training adaptations.

### Aerobic training response.

**Figure 3.**
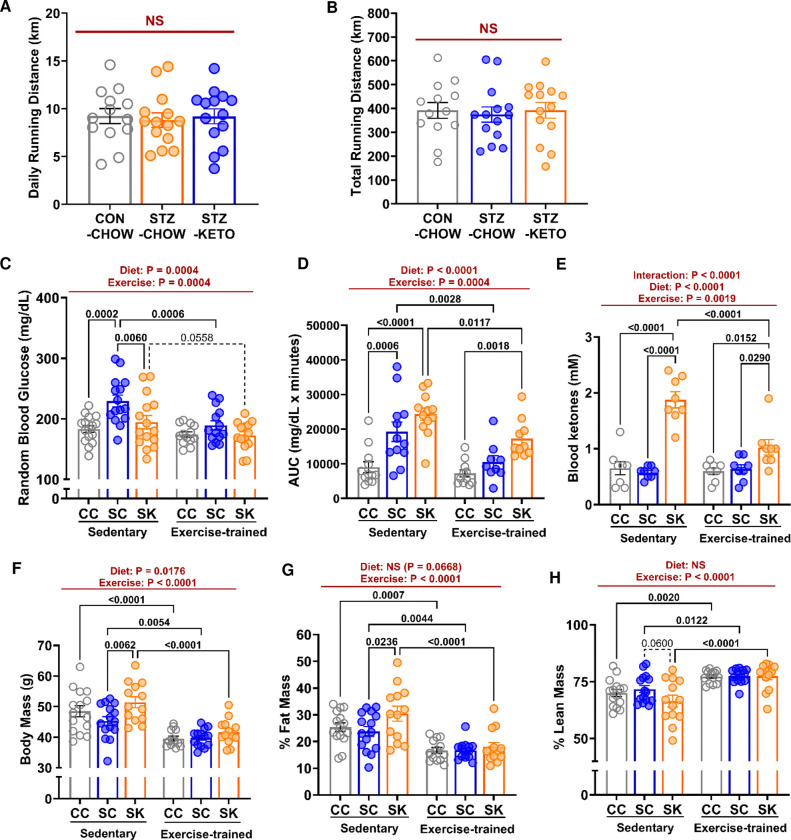
Exercise training improved health-related metabolic parameters similarly among all diet treated groups. **[A]** Daily running distance, and **[B]** total running distance over the training period were similar among groups. **[C]** Training improved random blood glucose and **[D]** glucose tolerance in all groups. **[E]** Blood ketones were decreased by training, but still remained higher in STZ-KETO. **[F]** Body mass was higher in sedentary STZ-KETO, but was reduced to the level of controls by exercise-training. **[G]** Fat mass was higher in sedentary STZ-KETO, but was reduced to the level of controls with exercise-training. **[H]** Percent lean mass was increased by exercise training in all groups. Results are displayed as mean ± SEM. Panels A and B were analyzed by 1-way ANOVA. Panels C-H were

To determine the effect of KETO on the response to aerobic training in STZ, mice were randomly allocated to a cage with (Exercise-Trained) or without (Sedentary) a running wheel. Daily (Figure 3A) and total (Figure 3B) running distance was similar among groups, demonstrating equal training dose. There was a main effect of exercise training to decrease blood glucose (Figure 3C). Glucose tolerance was also improved by exercise, but remained impaired in STZ-KETO compared to chow-fed controls (Figure 3D). Ketones were significantly decreased by exercise training (Figure 3E), but remained higher in STZ-KETO than chow-fed groups (Figure 3E). After 16-wks of dietary treatment, sedentary STZ-KETO displayed increased body mass associated with fat mass gain compared to STZ-CHOW (Figure 3F-G). In contrast, body mass and fat mass were similar in all exercise-trained groups, indicating that training can prevent fat-mass gains due to KETO observed in sedentary mice. Exercise training increased percent lean mass to a similar extent in all groups (Figure 3H). Overall, these data demonstrate that all groups received a similar training stimulus, and exercise produced the expected positive metabolic adaptations in all groups, regardless of glycemic status or diet.

**Figure 4.**
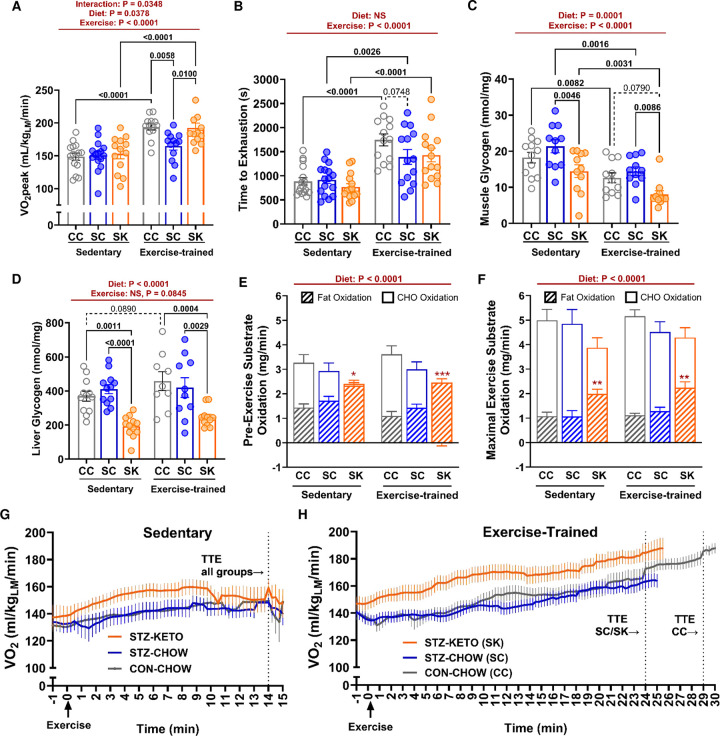
A ketogenic diet restores improvements in VO2peak with training in STZ-treated mice. **[A]** Peak aerobic capacity (VO2peak) was measured in exercise-trained and age-matched sedentary mice using the GXTm protocol. **[B]** Time to exhaustion (TTE) was recorded during the test as a measure of performance. **[C]** Muscle and **[D]** liver glycogen content were significantly reduced in mice consuming a ketogenic diet. Fat oxidation rates were higher in KETO-fed mice before exercise **[E]** and during the last two minutes of maximal exercise **[F]**. Conversely, maximal fatty acid oxidation rates were significantly higher in KETO-fed mice. VO2 kinetics during the GXTm protocol are shown for sedentary **[G]** and exercise-trained **[H]** mice. The mean TTE for each group is indicated by a dashed line to indicate the approximate end of testing. Data were analyzed by 2-way ANOVA with Tukey post-hoc testing. *P<0.05, **P<0.01, ***P<0.001 for fat oxidation vs CC and SC.

We have previously demonstrated that STZ-treated mice have blunted improvements in the key health marker of VO_2_peak with training, despite having other positive metabolic adaptations in response to training^1, 2^. To determine whether impaired improvements in VO_2_peak are prevented by a ketogenic diet, we performed maximal exercise capacity (VO_2_peak) and performance (Time to Exhaustion; TTE) testing in sedentary and exercise-trained mice. As differences in fat mass were observed among groups (Figure 3G), VO_2_peak was expressed per lean mass to minimize the influence of this potentially confounding variable. VO_2_peak was similar among sedentary groups (Figure 4A). In contrast, there were significant differences in VO_2_peak among groups that underwent exercise-training. Compared to CON-CHOW, STZ-CHOW had blunted improvements in VO_2_peak with training. However, a ketogenic diet restored exercise training-induced improvements in VO_2_peak in STZ (Figure 4A). All groups significantly improved exercise performance (TTE) with training (Figure 4B). However, unlike VO_2_peak, TTE was not significantly higher after training in STZ-KETO compared to STZ-CHOW. These data demonstrate that glucose lowering with a ketogenic diet is associated with improvements in the key health marker of VO_2_peak with training. Moreover, we demonstrate a disconnect between exercise capacity and performance in STZ-treated mice consuming a ketogenic diet.

### Nutrient storage in muscle and liver.

Skeletal muscle makes important contributions to whole-body aerobic capacity and exercise performance. To determine potential mechanisms for higher VO_2_peak in the absence of improved performance in STZ-KETO, we examined several aspects of muscle metabolism and phenotype. To enhance aerobic performance, many athletes “carbohydrate load” to increase muscle and liver glycogen stores prior to competition. Indeed, muscle glycogen plays an important role in exercise performance- especially during high-intensity exercise of moderate duration such as our aerobic testing protocol^30^. To determine whether glycogen availability may have impacted performance, we measured muscle and liver glycogen in sedentary and exercise-trained mice. We demonstrate main effects of both diet and exercise to reduce muscle glycogen concentration (Figure 4C). The lowest muscle glycogen was observed in trained STZ-KETO, where glycogen levels were half the concentration of STZ-CHOW. Liver glycogen was also significantly lower in STZ-KETO, irrespective of training status (Figure 4D).

In addition to glycogen, stored triacylglycerol, which is also a key substrate for aerobic exercise, was measured in muscle and liver. In contrast to glycogen, there was no difference in muscle triglyceride (glycerol) storage in STZ-KETO, but liver triglycerides were significantly higher in mice fed a ketogenic diet (Supplementary Figure 2A & B). Overall, our data demonstrate notable changes in muscle and liver nutrient storage in response to a ketogenic diet. Our results point toward lower glycogen availability as a possible mechanism for a lack of improvement in exercise performance in trained STZ-KETO compared to STZ-CHOW, despite having significantly higher VO_2_peak. Our data also indicate a shift toward fat storage as a fuel in STZ-KETO.

### Substrate oxidation during maximal exercise.

Altered substrate storage in muscle and liver may contribute to altered substrate selection during maximal exercise testing. Therefore, we determined fat and carbohydrate oxidation rates calculated by indirect calorimetry before, and during the last two minutes of maximal exercise testing. Hyperglycemia in STZ-CHOW did not alter substrate utilization at rest (Figure 4E) or during maximal exercise (Figure 4F) compared to CON-CHOW. In contrast, STZ-KETO had significantly higher fatty acid oxidation rates compared to CHOW-fed mice under both conditions. At rest, STZ-KETO relied solely on fat as a substrate. In contrast to CHOW-fed mice, used a mixture of fat and carbohydrate (Figure 4E). During maximal exercise, carbohydrate oxidation rates increased in all groups (Figure 4F), as expected. However, KETO maintained ~2-fold higher rates of fatty acid oxidation compared to CHOW-fed mice. Notably, fat oxidation rates in STZ-KETO were similar in sedentary and exercise-trained mice. As trained STZ-KETO had significantly higher VO_2_peak, this indicates that improved VO_2_peak with training is independent of enhanced fatty acid oxidation. Higher fatty acid oxidation rates in STZ-KETO were accompanied by a lower respiratory exchange ratio (RER) at rest and during maximal exercise (Supplementary Figure 2C).

Higher reliance on fatty acids for fuel can increase oxygen consumption rates due to greater oxygen demand of fatty acid vs. carbohydrate oxidation. Consistent with higher rates of fatty acid oxidation, our data demonstrate higher oxygen consumption during the first 10 minutes of maximal exercise testing in STZ-KETO. In sedentary mice, elevated oxygen consumption in STZ-KETO was not sustained at higher exercise intensities, resulting in a similar VO_2_peak in all sedentary groups (Figure 4G). However, in trained STZ-KETO, VO_2_ remained higher than STZ-CHOW until exhaustion (Figure 4H). Taken together, our data demonstrate that a ketogenic diet alone does not impact VO_2_peak in hyperglycemic mice. However, when combined with aerobic exercise training, a ketogenic diet can restore improvements in VO_2_peak that are blunted in hyperglycemic mice, signifying a diet-training interaction that is independent of fatty acid oxidation rates.

### Substrate oxidation and circulating metabolites with moderate exercise.

**Figure 5.**
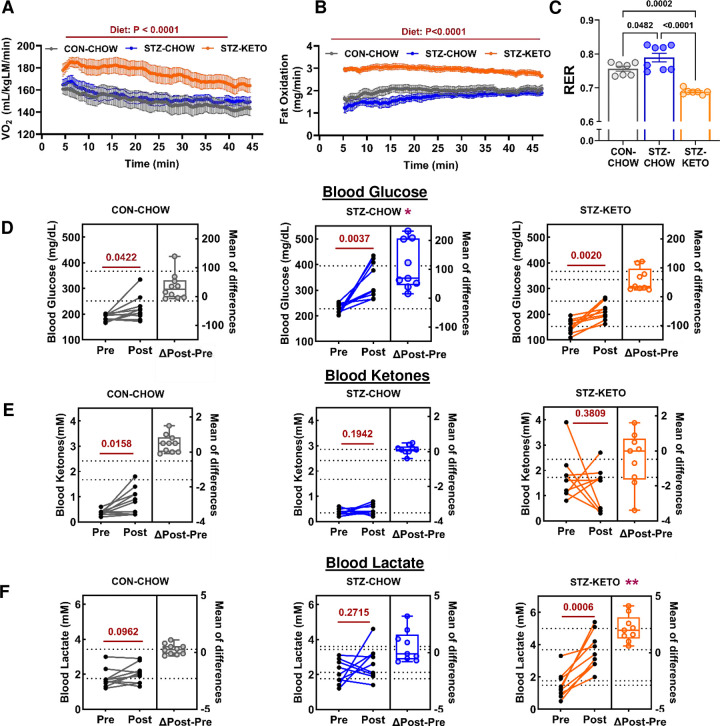
A ketogenic diet alters fuel selection and blood metabolite levels during moderate exercise in STZ-treated mice. **[A]** Oxygen consumption (VO2) was measured during a 45-minute bout of moderate intensity (~60% VO2peak) treadmill running exercise. Data from the steady state (5–45 min) exercise period are shown. **[B]** Fat oxidation was calculated over the same acute exercise period. **[C]** The mean Respiratory exchange ratio (RER) was significantly lower in STZ-KETO vs chow-fed controls throughout exercise, indicating a higher reliance on fatty acid oxidation during exercise. Blood glucose **[D]**, ketones **[E]**, and lactate **[F]** were measured before and after the exercise bout, and the change in metabolite levels were calculated. Panels A and B were analyzed by 1-way ANOVA. Panels C-F were analyzed by paired t-test to determine significant changes in response to exercise. *Indicates P<0.05 for the ΔGlucose of STZ-CHOW vs. CON-CHOW. **Indicates P<0.01 for ΔLactate of STZ-KETO vs. CONCHOW. N=7–8/group.

Maximal exercise to exhaustion is helpful to determine key metabolic parameters such as VO_2_peak. However, maximal exercise is bioenergetically distinct from moderate aerobic exercise, which better reflects a “typical” session of aerobic exercise performed by the general population. Therefore, we investigated oxygen consumption and substrate utilization in a separate cohort of sedentary mice that underwent 45 minutes of moderate (~60% VO_2_peak) steady-state treadmill running exercise. This exercise intensity normally results in a mixture of carbohydrate and fat oxidation by skeletal muscle. Oxygen consumption was higher throughout exercise in STZ-KETO compared to STZ-CHOW and CON-CHOW, despite the exercise being of a similar relative intensity in all groups (Figure 5A). Increased oxygen consumption in STZ-KETO was likely due to enhanced fat oxidation during exercise, as STZ-KETO demonstrated significantly higher fatty acid oxidation rates throughout the steady-state exercise bout (Figure 5B). In support of this, the respiratory exchange ratio (RER; VCO_2_/VO_2_) was ~0.68 in STZ-KETO, indicating a complete reliance on fatty acid oxidation during moderate exercise (Figure 5C). RER values less than 0.7 may indicate ketogenesis without subsequent ketone oxidation^31^. In contrast, RER was significantly higher in chow-fed animals, with the highest RER occurring in STZ-CHOW, likely due to increased circulating glucose availability in this group. Thus, our data indicate that there are differences in substrate utilization during moderate exercise among groups, with fat oxidation rates showing the following hierarchy: STZ-KETO>CON-CHOW>STZ-CHOW.

The profound alterations in substrate oxidation that we observed with KETO may also impact utilization and/or synthesis of circulating metabolites during exercise. To investigate this, we determined the circulating levels of key metabolites before and after moderate exercise. Blood glucose was significantly increased post-exercise in all groups, although the increase was significantly higher in STZ-CHOW (Figure 5D). Blood ketones were only increased by exercise in CON-CHOW (Figure 5E). Ketones were maintained within a narrow margin before and after exercise in STZ-CHOW. In contrast, STZ-KETO showed a high variation of blood ketones at rest and after exercise. Interestingly, those mice with higher starting ketones tended to demonstrate decreased blood ketones after exercise, and vice versa. Blood lactate was unchanged by exercise in CON-CHOW and STZ-CHOW, which is characteristic of moderate intensity exercise. However, lactate was significantly increased after exercise in STZ-KETO leading to post-exercise lactate levels that were higher than chow-fed groups (Figure 5F). While lactate was historically considered a waste product of exercise, recent data have identified it as a key energetic substrate^32^. Taken together, our results demonstrate that a ketogenic diet in STZ-treated mice induces an increase in oxygen consumption and fatty acid oxidation, while altering the levels of key circulating substrates such as glucose and lactate during moderate-intensity exercise.

### Skeletal muscle glucose regulation.

**Figure 6.**
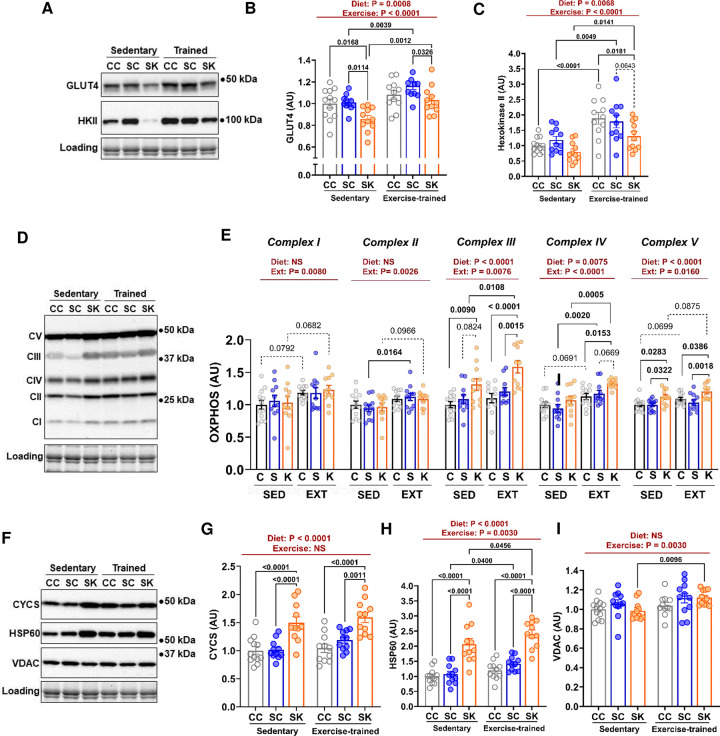
Downregulation of glucose metabolism markers and upregulation of mitochondrial protein markers by a ketogenic diet. Protein lysates of pulverized gastrocnemius muscles were analyzed by Western blotting and relative protein levels were quantified. **[A]** Muscle GLUT4 **[B]** and Hexokinase II **[C]** content were up-regulated by exercise, but demonstrated lower levels in mice fed a ketogenic diet. **[D]** In contrast, OXPHOS complex markers **[E]** were generally increased by a ketogenic diet, with complexes III, IV, and V also being upregulated by training. **[F]** Mitochondrial markers Citrate Synthase (CYCS; **[G]**) and HSP60 **[H]** were upregulated by a ketogenic diet, while VDAC **[I]** only demonstrated a main effect of exercise-training. Results are displayed as mean ± SEM. Statistical significance was determined by two-way ANOVA with Tukey Post-hoc testing. Main effects of diet and exercise are shown above each graph. Differences between groups are indicated by P-values.

Our data demonstrate significant alterations in substrate storage and utilization, and aerobic exercise adaptation in STZ-treated mice consuming a ketogenic diet. However, the mechanisms underlying these physiological changes are unknown. To determine how skeletal muscle adaptations may contribute to altered metabolism observed among groups, we examined several aspects of skeletal muscle phenotype. First, we assessed protein markers of glucose and fatty acid metabolism in skeletal muscle. Our data demonstrate significant, but divergent, main effects of diet and exercise-training on muscle GLUT4 and HKII content (Figures 6A-C). KETO decreased skeletal muscle levels of GLUT4 and HKII in both sedentary and exercise-trained mice. Down-regulation of these key glucose metabolism regulators in STZ-KETO is consistent with our observed reduction in glucose utilization and glycogen storage in this group. In contrast, exercise-training increased GLUT4 and HKII in STZ-treated mice, irrespective of diet. Notably, a ketogenic diet had a larger magnitude of impact on markers of glucose metabolism than exercise-training.

### Skeletal muscle mitochondrial protein markers.

Exercise typically results in higher mitochondrial density in muscle. Moreover, a higher reliance on fatty acid oxidation observed in both sedentary and trained STZ-KETO may be facilitated by changes in mitochondrial content or capacity. Indeed, our data demonstrate a main-effect of exercise training to increase protein levels of mitochondrial OXPHOS complexes I-V (Figure 6D-E). We also demonstrate a main effect of KETO to increase OXPHOS complexes III, IV, and V, independent of exercise-training. The increase in OXPHOS proteins in response to KETO was ~3-fold higher in magnitude than the effect of exercise-training, which increased OXPHOS by a modest, but reproducible ~10%. To further determine the effect of a ketogenic diet on muscle mitochondria, we quantified the relative levels of other key mitochondrial markers (Figure 6F). KETO induced a significant upregulation of citrate synthase (Figure 4G; 1.5-fold increase), and HSP60 (Figure 4H; 2-fold increase). In contrast, VDAC, which is also a mitochondrial marker, did not display a main effect of diet, but was significantly increased by exercise in STZ-KETO. Taken together, our data demonstrate multiple adaptations in skeletal muscle in response to consumption of a ketogenic diet that may underlie our whole-body observations of increased oxygen consumption, fatty acid oxidation, and reduced carbohydrate storage.

### Muscle mitochondrial morphology.

**Figure 7.**
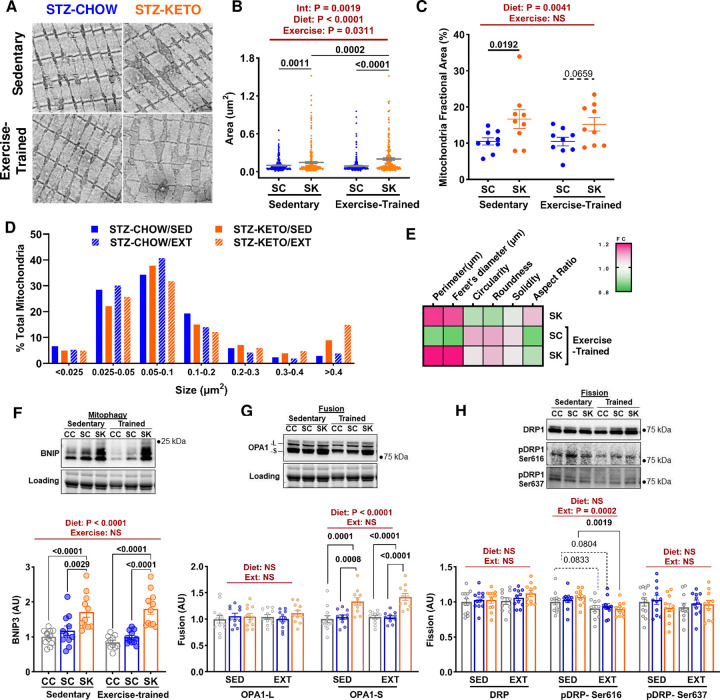
Ketogenic diet induced mitochondrial remodeling in STZ-treated mice. **[A]** Transmission electron microscopy was used to visualize and quantify plantaris muscle mitochondria in sedentary and exercise trained STZ-treated mice. **[B]** Mitochondrial size (area in μm^2^) was significantly larger in KETO-fed mice. Exercise-training and KETO had synergistic effects on mitochondrial size. **[C]** Fractional mitochondrial area (area of mitochondria/total muscle area) was significantly larger in KETO, but was not impacted by exercise-training. **[D]** Mitochondrial size distribution was altered by KETO with a shift toward very large (>0.4 μm^2^) mitochondria. **[E]** A heat map demonstrates quantification of other mitochondrial morphology parameters normalized to sedentary STZ-CHOW. Mitochondrial perimeter and Feret’s diameter were significantly larger in KETO. Protein markers of mitophagy **[F]**, and fusion **[G]** were increased by KETO. **[H]** The fission marker DRP1 was not impacted by KETO, but its phosphorylation at Ser616 was reduced by exercise. Results for A-E are representative of N=3 intermyofibrillar regions from N=3 mice/group. N=11–12 for panels F-G. Statistical significance determined by Two-way ANOVA followed by Tukey’s posthoc testing.

Our data demonstrate significant and exercise-independent increases in several muscle mitochondrial markers with KETO. To more directly assess mitochondrial content and morphology, we performed transmission electron microscopy (EM; Figure 7A). Our data demonstrate a significant increase in mitochondrial size in STZKETO compared to STZ-CHOW (Figure 7B). There was a synergistic effect of KETO and exercise to increase mitochondrial size, with only KETO-fed mice displaying an increase with training (Figure 7B). Mitochondrial density (% mitochondrial area per muscle area) was also larger in STZ-KETO, but was not impacted by exercise (Figure 7C). Mitochondrial size distribution shows a shift toward very large (>0.4 μm^2^) mitochondria in KETO-fed mice (Figure 7D). A heat map normalized to sedentary STZ-CHOW illustrates increase in mitochondrial perimeter and Feret’s diameter with KETO (Figure 7E). Quantification of these and other indices of mitochondrial morphology are shown in Supplementary Figure 3.

### Mitochondrial dynamics.

Changes in mitochondrial morphology are mediated by mitochondrial dynamic markers that regulate processes such as mitophagy, fusion, and fission. To determine potential mechanisms underlying the prominent changes in mitochondrial morphology that we observed in STZ-KETO, we quantified protein levels of mitochondria dynamic markers in skeletal muscle. BNIP3, a marker of mitophagy, was significantly increased in STZ-KETO, with no additional impact of exercise-training (Figure 7F). Similarly, the short form of OPA1, a marker of mitochondrial fusion, was significantly increased in STZ-KETO, with no additional impact of exercise-training (Figure 7G). Phosphorylation of DRP1 at Ser616, a marker of mitochondrial fission, was decreased by exercise, but not impacted by diet (Figure 7H). When considered collectively, our EM and protein data demonstrate that a ketogenic diet induces mitochondrial remodeling favoring mitophagy and fusion, resulting in larger mitochondria and a higher mitochondrial density in muscle.

### Aerobic remodeling of skeletal muscle.

**Figure 8.**
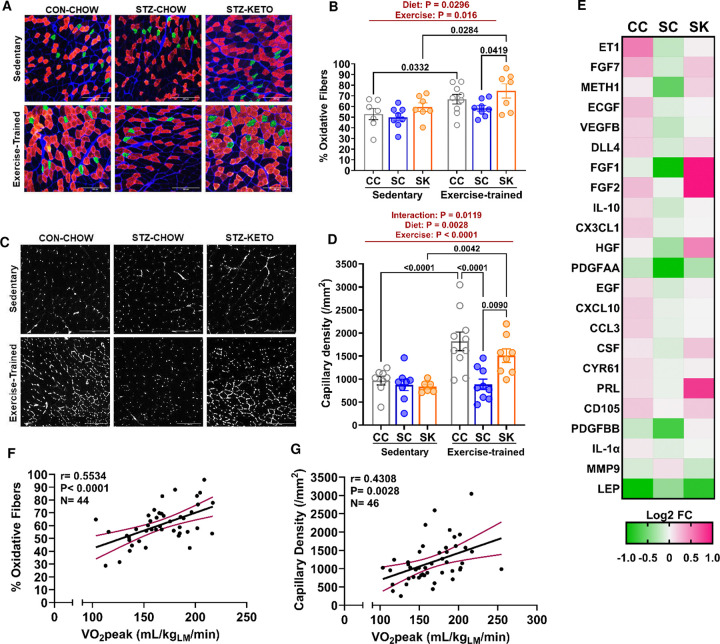
A Ketogenic diet restores aerobic muscle remodeling with exercise in STZ-treated mice. **[A]** Gastrocnemius muscle sections were stained for type 1 (green) and type 2A (red) myosin heavy chain. The extracellular matrix was stained with wheat germ agglutinin (blue). Images of the red (oxidative) portion of the gastrocnemius are shown. **[B**] The proportion of oxidative (type 1 + type 2A) fibers was quantified in the red gastrocnemius of Sedentary and Exercise-trained mice. **[C]** Capillaries in gastrocnemius sections were stained using fluorescently labeled Griffonia Lectin. **[D]** Capillary density was quantified and expressed per mm^2^ of muscle area. **[E]** Serum from exercise trained mice was used to quantify the relative protein levels of angiogenic regulators using a protein array. Relative expression normalized to sedentary controls is shown in a heat map. Pearson correlation analysis was performed to determine the relationship between whole-body VO_2_peak and **[F]** the percent of oxidative fibers in gastrocnemius muscle, or **[G]** muscle capillary density. Panels [B] and [D] were analyzed by two-way ANOVA followed by Tukey post-hoc testing with N=7–8/group.

Our data demonstrate significant effects of a ketogenic diet to increase muscle mitochondrial density and enhance fatty acid oxidation *in vivo*, independent of exercise-training. In contrast, KETO enhances VO_2_peak in STZ-treated mice only when combined with exercise training, indicating that mitochondrial function is likely not limiting for improvements in VO_2_peak with training in hyperglycemic mice. In addition to increased mitochondrial density, a more oxidative muscle fiber type and increased muscle capillary density are good predictors of high VO_2_peak in humans^33^. Moreover, our previous studies report that blunted improvements in VO_2_peak with training in hyperglycemia are associated with lower muscle capillary density and a more glycolytic muscle fiber-type^1, 2^, identifying these morphological markers in muscle as potential mechanisms for low VO_2_peak. To determine whether glucose-lowering with a ketogenic diet can restore training-induced remodeling of skeletal muscle we measured muscle fiber-type and capillary density by immunofluorescence staining (Figure 8). Main effects of diet and exercise were observed on muscle fiber-type, with the proportion of oxidative (Type 1 + 2A) fibers being higher in STZ-KETO compared to STZ-CHOW after training (Figure 8A & B). STZ-CHOW was the only group that did not demonstrate a significant increase in oxidative fiber proportion with training, indicating blunted exercise-induced muscle remodeling (Figure 8B). Our data demonstrate this remodeling can be restored with consumption of a ketogenic diet.

Skeletal muscle capillary density displayed main effects of exercise and diet, in addition to a significant interaction by two-way ANOVA (Figure 8C & D). Hyperglycemic mice (STZ-CHOW) had no significant increase in muscle capillary density with training; however, improved capillary density with training was restored in STZ-KETO (Figure 8D). Notably, the pattern for exercise-induced increases in capillary density mirrored our observed changes in VO_2_peak, with diet-exercise interactions observed for both phenotypes (Figure 4A). These data indicate that KETO may enhance exercise-induced angiogenesis, which appears to be impaired in hyperglycemic mice^2^. An increase in the capacity for muscle oxygen delivery due to increased capillary density may contribute to increased VO_2_peak in KETO-fed mice.

### Circulating angiogenic regulators.

Exercise-induced changes in capillary density may be due, in part, to changes in circulating angiogenic regulators that are upregulated by exercise-training. To determine whether KETO altered angiogenic regulators in exercise-trained mice, we performed a Proteome Profiler array of key proteins involved in tissue angiogenesis. Our analysis used pooled serum samples from each exercise-trained group normalized to sedentary normoglycemic controls. This assay detected 52 circulating angiogenic regulators in the serum from all experimental groups. Compared to sedentary CON-CHOW, exercise-training up- or down-regulated 16/52 (31%) of quantified proteins by 20% or more. Supplementary Table 1 shows the relative expression of all detected markers from each group. Targets demonstrating >20% up- or down-regulation between exercise-trained controls (CON-CHOW) and hyperglycemic mice (STZ-CHOW) are shown in a heat map to demonstrate changes in circulating angiogenic markers due to hyperglycemia (Figure 8E). Compared to CON-CHOW our data demonstrate down-regulation of several angiogenic proteins after training in the serum from hyperglycemic mice (STZ-CHOW; Figure 8E). Consistent with improved angiogenesis, KETO reversed many of the changes observed in angiogenic markers with hyperglycemia. Indeed, heat map analysis demonstrates more similar profiles in CON-CHOW and STZ-KETO, compared to STZ-CHOW. Proteins that demonstrated the highest upregulation with KETO include FGF2, FGF1, and Prolactin, all of which can have pro-angiogenic actions^34, 35^. Our data demonstrate that a ketogenic diet, when combined with exercise training, can restore the levels of circulating angiogenic markers and increases in muscle capillary density in hyperglycemic mice.

### VO_2_peak muscle phenotype associations.

A more oxidative fiber type and higher capillary density in muscle are strongly associated with VO_2_peak in humans. To determine whether fiber-type and capillary density are associated with improved VO_2_peak in our models, we performed linear regression and correlation analysis. Our data demonstrate a significant correlation (r=0.5534; P<0.00001) between VO_2_peak and the proportion of oxidative fibers in skeletal muscle (Figure 8F). VO_2_peak was also significantly correlated (r=0.4308; P=0.0028) with muscle capillary density (Figure 8G). Thus, enhancements in muscle remodeling with exercise are potential mechanisms for restored improvements in VO_2_peak with a ketogenic diet.

### Summary and conclusions.

**Figure 9.**
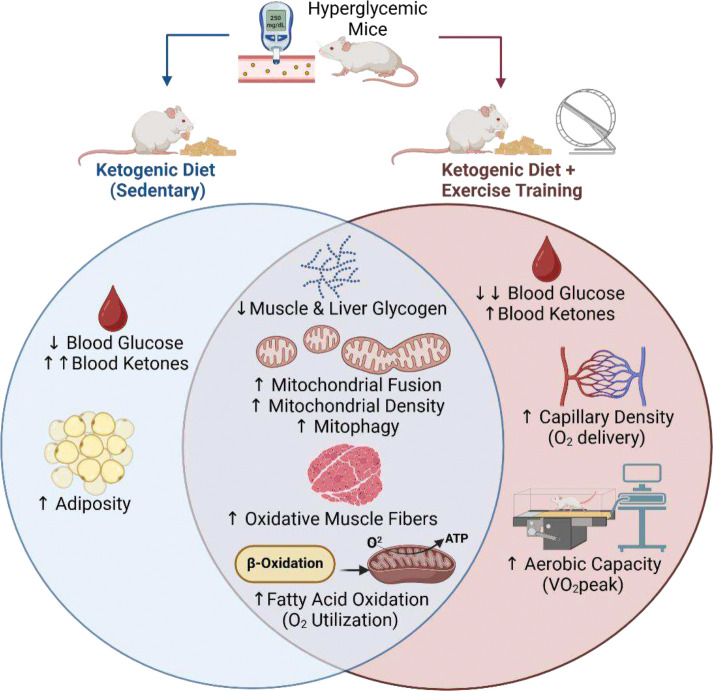
A summary of the effects of a ketogenic diet alone, or a ketogenic diet in combination with aerobic exercise training in hyperglycemic mice. Both treatment groups had reduced muscle and liver glycogen stores, higher muscle mitochondrial density and size, and a larger proportion of oxidative muscle fibers. The ketogenic diet (KETO) also increased rates of fatty acid oxidation at rest and during exercise- independent of exercise-training status. In the absence of exercise, KETO normalized blood glucose levels, increased blood ketones, and increased fat mass compared to chow-fed mice. When combined with exercise training, KETO-fed mice had even larger reductions in blood glucose, while KETO-induced increases in blood ketones were attenuated. In hyperglycemic mice, only those treated with KETO displayed exercise-induced increases in capillary density and VO_2_peak, demonstrating diet-training interactions. Figure was created using Biorender.com.

Our data demonstrate that a ketogenic diet can effectively normalize blood glucose levels in streptozotocin-treated mice with hyperglycemia. In conjunction with improved glycemia, increases in aerobic capacity (VO_2_peak) with training were restored by KETO. In the absence of training, KETO increased fatty acid oxidation rates, while down-regulating glucose regulatory proteins and glycogen storage in muscle and liver. In skeletal muscle, these shifts in substrate utilization were associated with striking changes in mitochondrial density and morphology, likely due to upregulation of key mitochondrial remodeling proteins such as BNIP3 and OPA1. When combined with training, KETO restored muscle remodeling processes that are blunted with hyperglycemia, including an increase in oxidative muscle fibers and capillary density. Our data identify a ketogenic diet as a potential treatment for impaired aerobic response to training that is associated with hyperglycemia (Figure 9).

## DISCUSSION

Hyperglycemia impacts an increasingly substantial proportion of the population, including those with type 1 diabetes, type 2 diabetes, insulin resistance, and other metabolic disorders^28, 29^. Regular physical exercise can improve metabolic health and reduce associated risks like cardiovascular disease. However, there is significant evidence that those with chronic hyperglycemia, regardless of its etiology, have a blunted cardiorespiratory response to aerobic training^3, 6, 36^. Peak oxygen consumption rate (VO_2_peak), which is a key predictor of health and longevity, is consistently lower in those with hyperglycemia, even when exercise training levels are matched^6, 7, 36^. Data from the present study support the hypothesis that hyperglycemia has a negative impact on aerobic exercise adaptation. Moreover, we demonstrate that normalizing blood glucose levels using a high-fat, low-carbohydrate diet can restore improvements in aerobic capacity (VO_2_peak) and skeletal muscle remodeling with training. Specifically, training-induced shifts in muscle toward a more oxidative fiber-type and increased capillary density are blunted in hyperglycemic mice, but these adaptations were normalized in STZ-treated mice consuming a ketogenic diet.

Investigations in human participants demonstrate increased fatty acid oxidation rates with consumption of high-fat, low-carbohydrate diets^37–39^. Similar to clinical investigations, we observed a notable increase in fatty acid oxidation rates both at rest and during exercise in STZ-KETO. Our data indicate that KETO can have similar metabolic effects in a model of hyperglycemia as are seen in healthy young humans. In addition, we demonstrate that KETO can induce a significant increase in muscle mitochondrial density and up-regulate key mitochondrial remodeling proteins such as BNIP and OPA1. Consistent with this, our electron microscopy analysis clearly illustrates an increase in mitochondrial size and a change in mitochondrial morphology with a ketogenic diet. When considered collectively, imaging and protein content analyses suggest a shift toward mitophagy and mitochondrial fusion in response to a ketogenic diet. Thus, our data identify potential mechanisms by which skeletal muscle adapts to increased fat availability in conjunction with carbohydrate restriction.

An important aspect of our study design is that we determined the effects of KETO in both sedentary and exercise-trained groups. Using this approach, we identified distinct metabolic adaptations to KETO that are exercise-dependent, and -independent. For example, increases in oxygen consumption, fatty acid oxidation, and altered mitochondrial morphology with KETO were independent of exercise training. In contrast, KETO only improved VO_2_peak and muscle capillary density in STZ-treated mice when combined with exercise-training. These data indicate that increased oxidative capacity is not sufficient to improve VO_2_peak in hyperglycemic mice. Rather, restoration of blunted exercise-induced increases capillary density was better associated with improvements in VO_2_peak in response to KETO. In other words, our data demonstrate that oxygen delivery to muscle is likely more limiting than oxidative capacity in determining VO_2_peak, at least under conditions of hyperglycemia where basal oxidative capacity is not impaired.

While the key health marker of VO_2_peak was enhanced by a combination of KETO and training in hyperglycemic mice, exercise performance was not improved compared to chow-fed mice. This may have implications for competitive athletes with hyperglycemia who wish to enhance aerobic fitness, while also maintaining peak performance. We identified low muscle glycogen content as a likely mechanism for the disconnect between VO_2_peak and time to exhaustion in KETO-fed mice. Indeed, lack of glycogen impairs performance even in highly fit elite athletes with superior aerobic capacity^40^. This may indicate that if athletes with hyperglycemia restrict carbohydrate to maintain euglycemia while training, their performance may benefit if carbohydrates are restored in the diet before and during competition. In support of this idea, a series of investigations by Burke and colleagues demonstrate that consumption of a high-fat, carbohydrate restricted diet does not have a negative impact on time-trial performance in normoglycemic elite athletes if the time-trial is preceded by one day of carbohydrate restoration^38, 41, 42^. Similarly, in young normoglycemic men fed a high-fat diet for 7-weeks, 1-week of carbohydrate restoration led to increased muscle glycogen and an ~18% increase in performance^43^. In that study, athletic performance was impaired by a high-fat diet without carbohydrate restoration^43^, which is consistent with our data from hyperglycemic mice. In contrast, several studies demonstrate no detriment to time trial performance with high-fat, low-carbohydrate diets, even without carbohydrate supplementation^44–47^. Divergent results among laboratories indicate effects on performance may be specific to dietary paradigms (macronutrient composition and duration), training status, and performance outcomes being measured. Clinical studies on athletes with hyperglycemia are needed to determine whether they can optimize training adaptation using high-fat, low carbohydrate diets, and maintain performance using strategic carbohydrate supplementation.

Given that hyperglycemia in animal models and humans is associated with poor adaptive response to aerobic training^1–3, 6^, we attribute the reversal of “exercise resistance” in STZ-KETO to glucose normalization. However, it is possible that increased circulating ketones may have contributed to the positive effects of KETO on training response. Ketones can promote angiogenesis and increase circulating angiogenic markers^48, 49^. Ketones are also thought to be beneficial for cardiac function and remodeling^50^. This is in line with our observation of increased muscle capillary density and circulating angiogenic markers with training in STZ-KETO. We have previously demonstrated that glucose normalization with the SGLT2 inhibitor, canagliflozin, can also restore muscle capillary density with training in a model of hyperglycemia^1^. Notably, canagliflozin produced a similar increase in circulating ketones as a ketogenic diet in the current study^1^. Thus, although hyperglycemia and its associated tissue level modifications (e.g., glycation and fibrosis) are known to impair angiogenesis^51^, we cannot rule out a potential positive effect of ketonemia on our observed improvements in muscle remodeling and aerobic capacity. Exogenous ketone supplementation in normoglycemic individuals does not appear to enhance exercise performance^52^. However, studies on ketone supplementation under conditions of hyperglycemia are warranted to dissect apart the roles of glucose lowering and ketonemia that occur with a ketogenic diet.

The resurgence in popularity of ketogenic diets in recent years has been a source of controversy^20^. This dietary paradigm represents a large shift from previous widespread recommendations to limit dietary fat intake in favor of carbohydrates^19^. Although ketogenic diets have well-documented benefits for weight loss and associated metabolic improvements in humans, there is concern that long-term consumption of such diets are unsustainable^53^. Indeed, although the diet in the present study was highly effective in normalizing glycemia, inducing mitochondrial remodeling, and enhancing aerobic adaptation in mice; consumption of a diet composed of 90% fat and 10% protein may not be practical for most people. We used strict restriction of carbohydrates in the present study to maximize metabolic perturbations. However, it is possible that inclusion of low glycemic index carbohydrates to a high-fat diet may improve dietary adherence while still allowing for tight glycemic control.

Another consideration regarding ketogenic diets is the potential impact of high dietary fat on cardiovascular risk factors^54^. This may be less of a concern in hyperglycemic individuals, as normalization of glycemia and improvement of VO_2_peak would act to reduce cardiovascular risk. In addition, we found that combining KETO with exercise training could minimize other potential negative effects on metabolic health, including increased adiposity and glucose intolerance. In contrast, our data demonstrate that increased liver triglycerides persisted in KETO-treated mice, despite exercise training. In humans, ketogenic diets have therapeutic benefits to reduce hepatic steatosis^55^, which may indicate this effect is specific to our mouse model and/or dietary paradigm. Nevertheless, cost-benefit analysis of a new dietary patterns must be considered for each individual. Our ketogenic diet was composed primarily of cocoa butter, which is relatively high in saturated fat. It is likely that altered fatty acid composition would also impact the potential health risks and benefits of a ketogenic diet.

A growing number of individuals who exercise regularly have hyperglycemia^56^, with some competing at high levels in their sport^57^. However, sports nutrition guidelines have been largely based on studies of normoglycemic individuals. Ketogenic diets have well-documented therapeutic benefits for many chronic diseases including obesity, diabetes, non-alcoholic fatty liver disease, and epilepsy. But, with respect to aerobic capacity and physical performance, it appears that ketogenic diets do not enhance or harm performance in elite athletes and healthy young individuals^21, 37, 58^. Here, we demonstrate that a ketogenic diet can enhance aerobic fitness when combined with exercise training in a mouse model of hyperglycemia. Thus, whether a ketogenic diet enhances muscle metabolism and function likely depends on individual circumstances and health history. In support of this, there is evidence that ketogenic diets can enhance muscle mass and function with age^59^ and muscular dystrophy^60^. Thus, individualized prescription of diet should be used to optimize the health benefits of exercise in different populations.

In summary, our investigation demonstrates that consumption of a ketogenic diet can enhance the adaptive response to aerobic training in a mouse model of hyperglycemia. Improvements in aerobic capacity with a ketogenic diet may be due, in part, to restored muscle remodeling with exercise, including a more oxidative fiber-type and increased capillary density. A ketogenic diet also had exercise-independent effects on mitochondrial phenotype, oxygen consumption, and fatty acid oxidation rates. Our results identify a ketogenic diet as a potential therapeutic strategy to improve aerobic capacity in those with hyperglycemia.

## MATERIALS AND METHODS

### Animal Experiments.

All mouse experiments were approved by the Institutional Animal Care and Use Committee of the Joslin Diabetes Center. Male CD1 mice (8 weeks old) were purchased from Charles River Laboratories. Female mice were not included as we and others have found they are resistant to induction of hyperglycemia by common methods, including diet^61^ and streptozotocin^62, 63^. While larger doses of streptozotocin can induce hyperglycemia in females, we found this leads to hepatic carcinogenesis, which has also been reported by others^64^. Therefore, in this case, females would not be appropriate to test our hypothesis. Mice were group-housed in a pathogen-free facility and maintained on a 12 h normal light/dark cycle with water and food ad libitum. Pre-treatment body weight and blood glucose were measured.

Following a 5-h morning (light cycle) fast, hyperglycemia was induced by intraperitoneal injection of 40 mg/kg streptozotocin (STZ, Tocris Bioscience, 1621) in Citrate Buffer, pH 4.5 (Boston Bioproducts, BB-2034) on 2 consecutive days. Control mice were injected with Citrate buffer. This STZ dosage has been shown to successfully induced moderate hyperglycemia without weight loss or severe complications in previous studies^1, 2^. All mice were fed with a low-fat control diet (CHOW; Research Diets, D10070802) consisting of 80% kcal carbohydrate, 10% kcal fat and 10% kcal protein during the post-inject period to assess development of hyperglycemia. Hyperglycemia, defined as random blood glucose >200 mg/dL, was established in STZ-injected mice within 2-weeks of injection. STZ-treated mice that did not reach the threshold for hyperglycemia were excluded.

Hyperglycemic mice were stratified to receive a ketogenic diet (STZ-KETO; Research Diets, D10070801) or continue receiving regular chow (STZ-CHOW). See Figure 1 for a graphical depiction of the timeline. The ketogenic diet consisted of 90% kcal from fat, 0% kcal carbohydrate and 10% kcal protein. Vehicle-treated normoglycemic controls were fed regular chow (CON-CHOW). Mice were continuously fed with assigned diet throughout the remaining 16 weeks of treatment, with weekly monitoring of body weight and blood glucose. Blood glucose, blood ketones, blood insulin, glucose tolerance and maximal exercise capacity testing were analyzed after 8 weeks of feeding to determine effect of ketogenic diet on hyperglycemia metabolic phenotypes.

### Voluntary Wheel Running Exercise.

Following 8 weeks of dietary treatment, mice from CON-CHOW, STZ-CHOW and STZ-KETO were randomly subdivided into sedentary or exercise-trained conditions. Exercise-trained mice were singly housed in a standard mouse cage equipped with a running wheel (Kaytee Run-Around Exercise Wheel, 100079365). Sedentary mice were housed in a similar cage without a running wheel, and were provided with alternative forms of enrichment. Running activity was recorded in 10 min intervals using a Hall Effect Sensor (0297–0501), Wheel Counter 8 Channel Interface (0297–0050) and a Quad CI-Bus to interface with CI Multi Device Software (v.1.5.5) from Columbus Instruments. Following 8 weeks of exercise training, blood glucose, blood ketones, glucose tolerance, body composition and maximal exercise capacity testing were measured to determine effect of exercise training on metabolism among treatment groups. Exercise-trained mice were withdrawn from wheel cages 24 hours prior to tissue collection to wash out the acute effects of exercise.

### Blood Parameters and Body Composition Measurement.

Blood was taken from tail vein of non-fasted mice in all measurements, unless otherwise stated. Blood glucose was measured at a similar time of the day using the INFINITY^®^ Blood Glucose Monitoring system (US Diagnostics). Blood ketones were measured using Precision Xtra Blood Glucose & Ketone Monitoring System (Abbott, 9881465). Blood lactate was measured using Lactate Plus Meter (Nova Biomedical, 62624). To measure serum insulin, mice were fasted overnight and blood was drawn from the tail vein into a centrifuge tube. Then, serum was separated by centrifugation at 3000g for 15 mins at 4°C. Serum insulin was analyzed by ELISA using a commercially available kit (EMD Millipore, EZRMI-13K). Body composition was measured in anesthetized mice by DEXA using a Lunar PIXImus2 mouse densitometer.

### Glucose Tolerance Tests.

Exercise-trained mice were withdrawn from wheel cages 24 hours before glucose tolerance measurement. The baseline glucose level was measured after a 5-hour morning (light cycle) fast. Mice were injected intraperitoneally with a glucose solution at a dose of 2 g glucose/kg body weight. Blood glucose was measured at 15, 30, 45, 60 and 120 min after glucose administration.

### Maximal Exercise Testing (VO_2_peak).

Exercise testing was conducted on Columbus or Sable Metabolic Treadmill systems (Sable Systems International, CS-003M-00). Oxygen and carbon dioxide were measured in the sealed treadmill chamber every 15 seconds. Prior to testing, mice were acclimatized for 2 consecutive days by placing them on the treadmill at rest for 10 minutes, followed by running at a low speed for 10 minutes. Exercise-trained mice were withdrawn from wheel cages 24 hours before testing. For maximal exercise capacity testing (VO_2_peak), graded maximal exercise testing (GTXm) protocol to exhaustion was used^65^. Exhaustion was defined as a mouse being in contact with the 1.1 mA electric shock grid for 5 continuous seconds. Researchers performing the testing were blinded to the experimental groups.

### Acute Exercise Protocol.

To determine effect of ketogenic diet on acute exercise response, a separate cohort of sedentary mice underwent a single steady-state exercise bout. CON-CHOW, STZ-CHOW and STZ-KETO mice (N = 9–10/group) underwent dietary treatment for 16-weeks prior to testing. The treadmill running protocol was performed at a 5° incline, at a speed of 11 m/min for 45 min. Our data indicate the intensity of this bout is approximately ~60–70% VO_2_max, thus representing a typical moderate aerobic exercise bout. Oxygen and carbon dioxide were measured every 15 seconds. Blood glucose, blood ketones and blood lactate were measured pre- and immediately post-exercise.

### Indirect Calorimetry.

Volume of Oxygen consumption (${\text{VO}}_{2}$), Carbon dioxide production (${\text{VCO}}_{2}$) and Respiratory exchange ratio (RER) was collected and derived from the MacroInterpreter program (Sable Systems International). Whole-body fat and carbohydrate oxidation were calculated from gas exchange before and during exercise with and assumption of negligible protein oxidation as shown below^66^.


$$\begin{array}{l} \text{Fat}\;\text{oxidation}\;\left(\text{mg}/\text{min}\right)=1.695\times {\text{VO}}_{2}-1.701\times {\text{VCO}}_{2}\\ \text{Carbohydrate}\;\text{oxidation}\;\left(\text{mg}/\text{min}\right)=4.210\times {\text{VCO}}_{2}-2.962\times {\text{VO}}_{2} \end{array}$$

### Glycogen assay.

An aliquot of ~10 mg pulverized gastrocnemius muscle was used to estimate muscle glycogen content. The muscle was hydrolyzed in 2N HCl for 2 hours at 95°C with constant agitation, followed by neutralization with 2N NaOH. The glucose concentration of the lysates was then calculated using hexokinase reagent (Cat#: G7517–120) in a 96-well plate. Glycogen content per muscle weight was calculated using absorbance values and dilution factors. For liver glycogen, approximately 20 mg of frozen tissue was used for the same protocol. However, reagent volumes for hydrolysis and neutralization were doubled to account for higher anticipated glycogen concentrations.

### Triglyceride Assay.

Aliquots of ~30 mg of skeletal muscle or liver were saponified overnight in ethanolic KOH at 55°C. Lysates were analyzed for glycerol content using a commercially available colorimetric assay (Cayman Chemical; #10010755) and triacylglycerol (glycerol content) was expressed per tissue weight.

### Immunoblotting.

Gastrocnemius muscle was pulverized with a liquid N_2_-cooled molar and pestle. An aliquot of pulverized muscle was homogenized in modified RIPA buffer (50 mM Tris-HCl pH 7.5, 10% Glycerol, 1% Triton-X, 0.50% Sodium Deoxycholate, 0.1% SDS, 1 mM DTT and 1X Protease/Phosphatase inhibitor (Thermo Fisher Scientific, 78444)) with a stainless steel bead using Qiagen Tissuelyser II. After centrifuging at 12000g, 4°C for 20 min, the supernatant was collected and protein concentration was measured by Bradford assay (Bio-Rad Laboratories, 5000006). Equal amounts of protein from each sample were mixed with Laemmli buffer and boiled at 95°C for 5 min, except for GLUT4 and OXPHOS which required unboiled proteins. Lysates were equally loaded on 4–15% Criterion^™^ TGX Stain-Free^™^ Protein Gel (Bio-Rad Laboratories, 5678085). Total protein loading was visualized on each gel using stain-free technology on the ChemiDoc^™^ Touch Imaging System (Bio-Rad Laboratories). Proteins were transferred to 0.2 μm nitrocellulose membranes using Trans-Blot Turbo Transfer System (Bio-Rad Laboratories, 1704150). Membranes were blocked using 5% non-fat dry milk in TBST at RT for 1 hour and incubated with primary antibodies (Table1) at 4°C overnight. Membranes were incubated with species-specific HRP-conjugated secondary antibodies and visualized by ChemiDoc^™^ Touch Imaging System. Intensity of protein bands were quantified by Image Lab software (Bio-Rad Laboratories).

### Immunofluorescence.

Gastrocnemius muscle was immediately frozen in liquid N_2_-cooled isopentane. Muscle was sectioned transversely at 6 μm thickness in a −20°C cryostat. For muscle fiber type staining, sections were incubated with primary antibodies against myosin heavy chain I (1:25; A4.840, DSHB) and myosin heavy chain IIa (1:25; SC-71, DSHB) in TBS + 1% BSA + 1% NGS overnight at 4°C. After rinsing, mouse IgG1 fluorescent conjugate (A21124; red) and mouse IgM (A21042; green) secondary antibodies were added at 1:1000 in 1% BSA in TBS + 1% NGS for 1 h. Wheat germ agglutinin (1:250; W7024, Invitrogen) was added with secondary antibodies for visualization of unstained fibers. For capillary staining, sections were fixed for 10 min with 10% Neutral Buffered Formalin (NBF) before staining. Fluorescent Griffonia lectin (1:100; FL12015, Vectorlabs) was applied for 1 h at room temperature. Slides were imaged using EVOS M7000 imaging system and quantified using ImageJ software^67^.

### Transmission Electron microscope (TEM).

Plantaris muscle was excised and immediately immersed in fixative buffer (1.25% formaldehyde, 2.5 % glutaraldehyde and 0.03% picric acid in 0.1 M Sodium cacodylate buffer, pH 7.4). Muscle was cut into an estimated 1 mm^2^ piece and incubated in fixative buffer at 4°C until imaging. Fixed samples were processed through a routine TEM preparation protocol at the Harvard Medical School Electron Microscopy Core Facility. Images were taken using a JEOL 1200EX transmission electron microscope. To analyze mitochondria, intermyofibrillar mitochondria (IFM) rich areas from STZ-CHOW and STZ-KETO with and without exercise training were captured in each sample at 3000x magnification. Images from 3 mice/group and 3 independent fields/mouse were quantified resulting in 9 images/group. The investigator performing quantification was blinded to the experimental groups. All analysis was performed using ImageJ software. Mitochondrial number and volume density were quantified from an area of ~88 μm^2^ for each image. Mitochondrial morphology including surface area, perimeter, Feret’s diameter, Circularity, roundness, solidity and aspect ratio were analyzed from individual mitochondria within 1 representative field from each group. The number of mitochondria analyzed for morphology from each group is: STZ-CHOW/SED = 291, STZ-KETO/SED = 311, STZ-CHOW/EXT = 290 and STZ-KETO/EXT = 268.

### Proangiogenic protein profiler analysis.

Mice were fasted for 2–4 hours during the early light cycle, and blood was collected by cardiac puncture under deep anesthesia. Samples were centrifuged at 2000g for 15 min at 4 °C to separate serum. Serum from exercise-trained CON-CHOW, STZ-CHOW, STZ-KETO and sedentary CON-CHOW were used to quantify mouse angiogenesis-related proteins by following the manufacturer’s protocol (R&D Systems, ARY015). Equal volumes of serum from 4 mice per group were pooled together and incubated on protein array membranes. Membranes were visualized with signal accumulation for 1– 10 minutes using a ChemiDoc^™^ Touch Imaging System. Each protein spot was quantified using Image Lab software (Bio-Rad Laboratories). Each detected angiogenesis-related protein from exercise-trained mice were normalized to sedentary CON-CHOW.

### Data and Statistical Analysis.

Data are presented as mean ± SEM otherwise stated. For pre-training data with three experimental groups, one-way analysis of variance (ANOVA) was used to compare effects between CON-CHOW, STZ-CHOW and STZ-KETO. For post-training data with six groups, two-way ANOVA was used to determine the main effects of diet and exercise training. Tukey multiple comparison tests were used for post hoc testing. For acute exercise studies, paired t-tests were used to compare differences in blood glucose, blood ketones and blood lactate levels pre- and post-exercise. Data are presented as connecting points of pre-post levels from individual mice with estimation plot demonstrating the delta values. One-way ANOVA was used to compare ΔPost-Pre exercise blood parameters between groups. Pearson correlation analysis was used to determine the relationship between maximal exercise oxidative capacity (VO_2_peak) and muscle remodeling markers.

## Supplementary Material

Supplement 1

## Figures and Tables

**Table 1:** Primary antibodies list

Primary Antibodies	Vendor	Cat no.	Dilution

*Western Blot Antibodies*			
OXPHOS cocktail	Abcam	ab110413	1:250
HKII	Cell Signaling Technologies	2867	1:1000
HSP60	Cell Signaling Technologies	12165	1:1000
CYTC	Cell Signaling Technologies	4280	1:1000
VDAC	Cell Signaling Technologies	4661	1:1000
GLUT4	Abcam	Ab654	1:1000
BNIP3	Cell Signaling Technologies	3769	1:1000
OPA1	Cell Signaling Technologies	80471	1:1000
DRP1	Cell Signaling Technologies	8570	1:1000
Phospho-DRP1 (Ser616)	Cell Signaling Technologies	4494	1:1000
Phospho-DRP1 (Ser637)	Cell Signaling Technologies	6319	1:1000
*Immunofluorescence Antibodies*			
Myosin heavy chain Type IIA	Developmental Studies Hybridoma Bank	SC-71	1:25
Myosin heavy chain Type I	Developmental Studies Hybridoma Bank	A4.840	1:25
Wheat Germ Agglutinin	Invitrogen	W 7024	1:250
Griffonia Simplicifolia Lectin I	Vector Laboratories	FL-1201-.5	1:100

## Data Availability

Data supporting the findings of this study are available in the primary figures, supplementary materials, or at FigShare https://doi.org/10.6084/m9.figshare.28191209.
