# Supplementary material for: A ketogenic diet enhances aerobic exercise adaptation and promotes muscle mitochondrial remodeling in hyperglycemic mice
Source: Res Sq. 2025 Jan 29:rs.3.rs-5814971. Preprint. [Version 1] doi: 10.21203/rs.3.rs-5814971/v1 (PMC11838742; doi:10.21203/rs.3.rs-5814971/v1)
Supplement: Supplement 1 [file NIHPPrs5814971v1-supplement-1.pdf]

SUPPLEMENTARY FIGURES

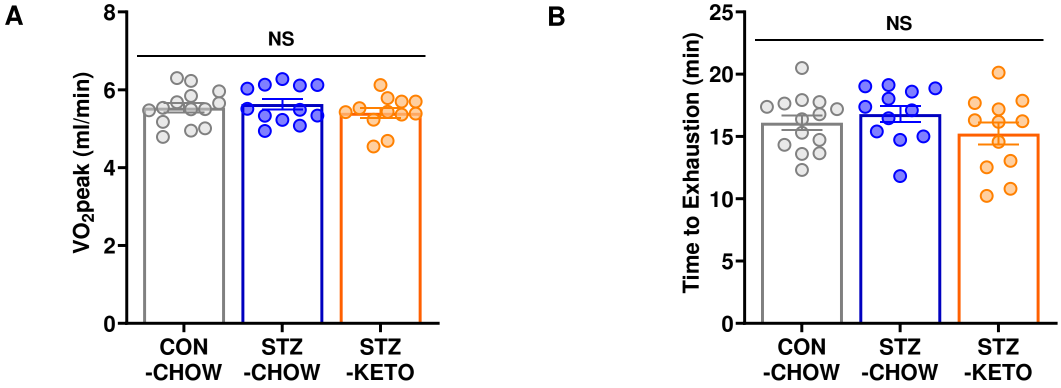

Supplementary Figure 1. Hyperglycemia was induced in CD-1 mice using streptozotocin (STZ). Normoglycemic controls were injected with citrate buffer vehicle (CON). Hyperglycemic mice received normal chow diet (CHOW) or a ketogenic diet (KETO). Following 8-wks of dietary treatment **[A]** VO_2peak and **[B]** time to exhaustion (TTE) during a graded exercise test were measured. Results are displayed as mean ± SEM. No significant differences were noted among groups by 1-way ANOVA.

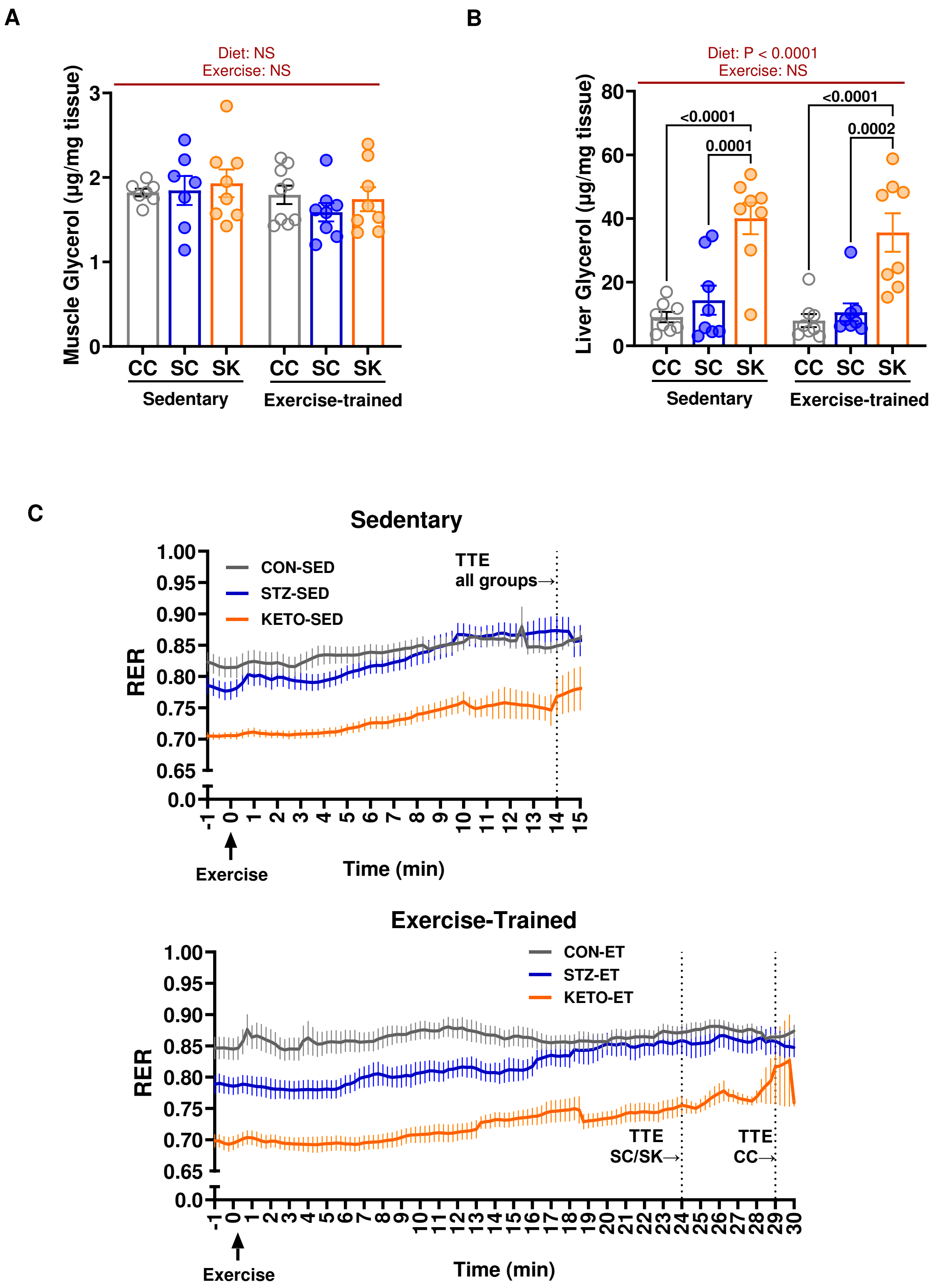

Supplementary Figure 2. Triacylglycerol (glycerol) concentration was measured in the **[A]** skeletal muscle, and **[B]** liver of sedentary and exercise-trained mice. **[C]** Respiratory exchange ratio was calculated during maximal exercise testing in sedentary and exercise-trained mice. CC, CON-CHOW; SC, STZ-CHOW; SK, STZ-KETO. Results are displayed as mean ± SEM. Differences among groups were determined by 2-way ANOVA with Tukey post-hoc testing.

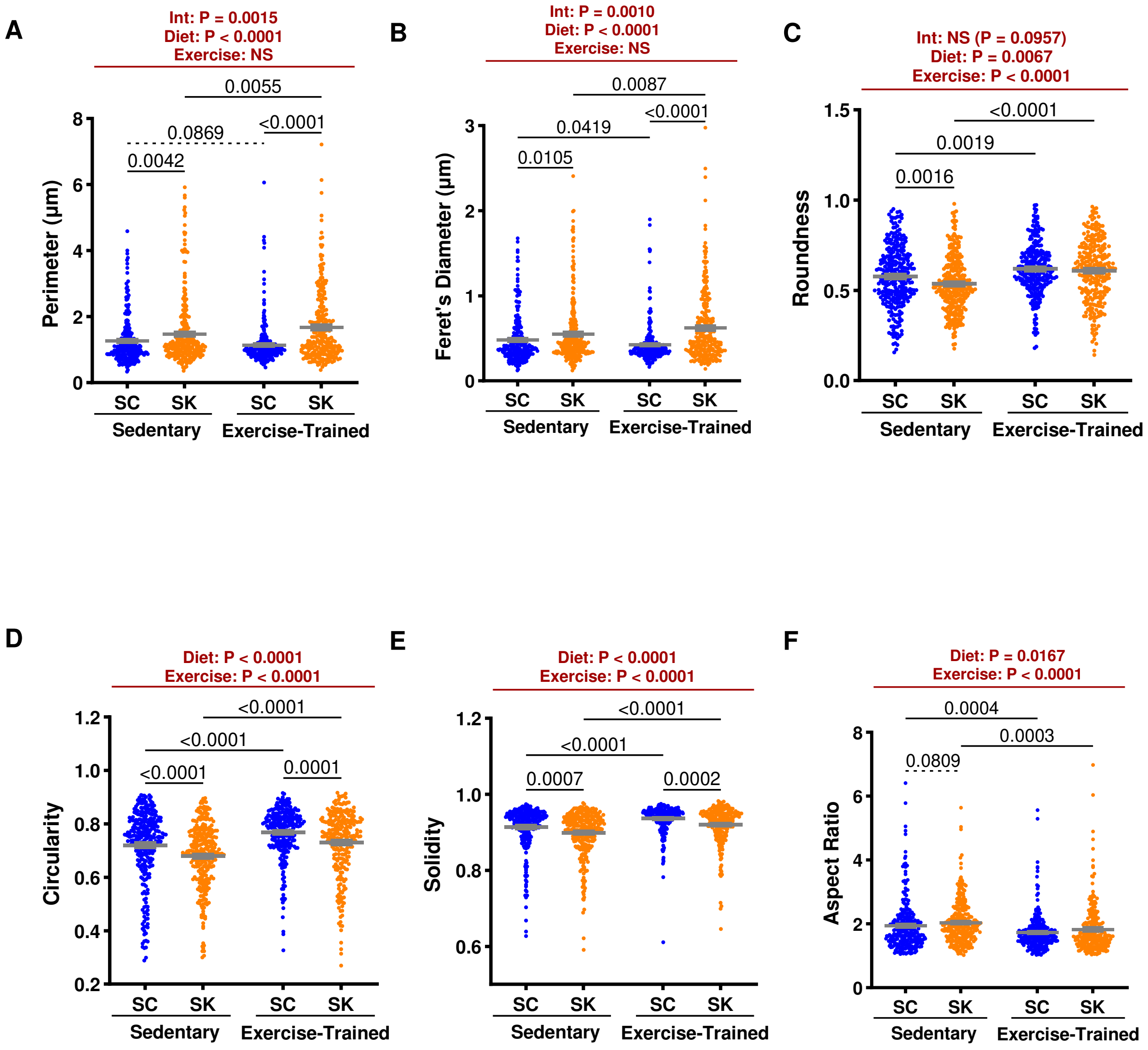

Supplementary Figure 3. Mitochondrial morphology was measured from TEM images of plantaris muscle collected from sedentary and exercise-trained STZ-CHOW (SC) and STZ-KETO (SK) mice. [A] Perimeter, [B] Feret's Diameter, [C] Roundness, [D] Circularity, [E] Solidity, and [F] Aspect ratio were assessed. Data represent intermyofibrillar mitochondria from N=3 mice per group. Data were analyzed by 2-way ANOVA with Tukey post-hoc testing. Each data point represents an individual mitochondrion.

Supplementary Files

This is a list of supplementary files associated with this preprint. Click to download.

- Supplementtable1.xlsx
